# CRISPR Cas9-guided chromatin immunoprecipitation identifies miR483 as an epigenetic modulator of *IGF2* imprinting in tumors

**DOI:** 10.18632/oncotarget.10918

**Published:** 2016-07-29

**Authors:** Yiqun Zhang, Ji-Fan Hu, Hong Wang, Jiuwei Cui, Sujun Gao, Andrew R. Hoffman, Wei Li

**Affiliations:** ^1^ Stem Cell and Cancer Center, First Affiliated Hospital, Jilin University, Changchun, Jilin 130061, P.R. China; ^2^ Department of Medicine, Stanford University Medical School, VA Palo Alto Health Care System, Palo Alto, CA 94304, USA

**Keywords:** IGF2 imprinting, tumor, epigenetics, histone K27 methylation, allelic expression

## Abstract

The normally imprinted insulin-like growth factor II (*IGF2*) gene is aberrantly upregulated in a variety of human malignancies, yet the mechanisms underlying this dysregulation are still poorly defined. In this report, we used a CRISPR Cas9-guided chromatin immunoprecipitation assay to characterize the molecular components that participate in the control of *IGF2* gene expression in human tumor cells. We found that miR483, an oncogenic intronic miRNA, binds to the most upstream imprinted *IGF2* promoter, P2. Ectopic expression of miR483 induced upregulation of *IGF2* expression, in parallel with an increase in tumor cell proliferation, migration, invasion, and tumor colony formation. miR483 induced loss of *IGF2* imprinting by altering the epigenotype at P2, with reduction in histone H3K27 methylation and a decrease in chromatin binding of two imprinting regulatory factors, CTCF and SUZ12. This study identifies a new role for miR483 in the regulation of *IGF2* gene expression through the alteration of the promoter epigenotype.

## BACKGROUND

Insulin-like growth factor II (IGF-II), a fetal mitogen with both growth promoting and metabolic effects, is dysregulated in a variety of human malignancies [1–3]. By binding to the type 1 IGF receptor (IGF1R) [4–6], IGF-II promotes tumor growth through autocrine, paracrine and endocrine pathways. By stimulating the MAPK and/or PI3-K/AKT signaling cascades, IGF-II causes reduced apoptosis, increased cell proliferation and drug resistance [7–10]. As a result, the IGF1R has been studied as a target for the development of tumor-specific gene therapy [11].

The gene encoding IGF-II, *IGF2*, is a maternally imprinted gene located on chromosome 11p15 [2, 12]. In postnatal life, four promotors regulate *IGF2* gene expression; promotor P1 directs biallelic expression, while promotors P2-P4 stimulate monoallelic expression of *IGF2* in most tissues except brain [13]. Monoallelic gene expression is regulated by allele-specific epigenetic modifications in the imprinting control region (ICR) located between *IGF2* and *H19* on chromosome 11p15.5 [14, 15]. Loss of *IGF2* imprinting (LOI) with biallelic expression of *IGF2* is a hallmark of many human tumors, especially childhood tumors [2, 3], and of cancer stem cells [16]. LOI has been associated with increased cellular proliferation and increased sensitivity of the IGF1R signaling pathway.

Little is known about the molecular mechanisms underlying the activation of the normally suppressed maternal *IGF2* allele in tumors with *IGF2* LOI. Reports regarding epigenetic modifications in the ICR are inconsistent [17, 18], and epigenetic modulators in the *IGF2* promoter regions have not been extensively studied. Therefore, we decided to identify molecular components in the major *IGF2* promoters that regulate *IGF2* expression. The CRISPR-Cas9 system has been used to genomically edit specific genes [19, 20]. When fused to transcriptional repressors or enhancers, the gRNA-guided enzyme can be used to modulate the activity of gene promoters [21–24]. We utilized a CRISPR Cas9-guided chromatin immunoprecipitation assay to pull down the *IGF2* promoter complex, and we identified miR483 as a molecule that interacts with the *IGF2* promoter and participates in the regulation of *IGF2* imprinting.

## RESULTS

### Identification of the binding of miR483 to the *IGF2* promoter

Loss of *IGF2* imprinting, a molecular hallmark of many tumors, is characterized by activation of the normally suppressed maternal promoters [25, 26]. We hypothesized that molecules that interact with the *IGF2* promoter, particularly noncoding RNAs, might be potential candidates for controlling *IGF2* allelic expression. We utilized a Cas9-guided chromatin immunoprecipitation assay to pull down candidate molecules that interact with the *IGF2* promoters. In this assay, a lentiviral vector containing the mutated Cas9 (dCas9) and two Cas9 gRNAs (Supplementary Figure 1) was stably transfected in target cells. dCas9 is a catalytically dead CRISPR Cas9 mutant, which is defective in DNA cleavage, but maintains the ability to bind to the gRNA-guided gene target [21, 27]. The binding specificity is determined by both gRNA-DNA base pairing and a short DNA motif (protospacer adjacent motif [PAM] sequence: NGG) juxtaposed to the DNA complementary region [28–30].

**Figure 1:**
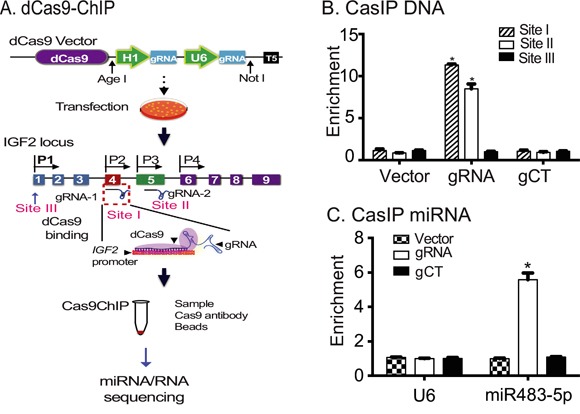
Identification of miR483 as a component of the *IGF2* promoter complex **A**. Diagram of the CRISPR Cas9-guided chromatin immunoprecipitation. dCas9: mutated Cas9; gRNA: Cas9 guiding RNA; U6: RNA polymerase III U6 promoter; *pIGF2: IGF2* promoters; CasIP: Cas9-guided immunoprecipitation. dCas9 lacks DNA cleavage activity, but is still able to bind to its target genes through a mechanism of base pairing between the gRNA and target DNA. After fixation, the dCas9-*IGF2* promoter chromatin complex was immunoprecipitated by an anti-Cas9 antibody. After removal of crosslinks, the captured miRNAs and RNAs were sequenced to identify the components that interact with the *IGF2* promoters (Sites I, II, III). **B**. The binding of dCas9-gRNA to *IGF2* promoters. Cells were transfected with dCas9-gRNA (gRNA), dCas9-gRNA control (gCT), and dCas9 vector control (Vector). After immunoprecipitation, the binding to the *IGF2* promoters was measured by PCR primers from Sites I, II at the imprinted promoters P2 and P3, and a non-target Site III at the non-imprinted promoter P1. Note the specific binding of Cas9-gRNA to the imprinted *IGF2* P2 and P3 sites. **C**. Identification of miR483 in the *IGF2* promoter complex. After CasIP, the captured miRNAs were reverse transcribed. Quantitative PCR was used to quantitate the abundance of miR483 in the Cas9-captured complex. Small nuclear RNA U6 was used as the internal control.

After selection, stable cells were treated with 1% formaldehyde to fix the dCas9-gRNA-*IGF2* promoter chromatin complex. A Cas9 antibody was then used to immunoprecipitate the Cas9-*IGF2* promoter chromatin complex. The components that interacted with the promoters, including putative modulators of *IGF2* imprinting, were eluted and identified by sequencing (Figure 1A).

In this study, we designed two guiding RNAs (gRNA) directed to *IGF2* promoters P2 and P3 (Site I and Site II), both of which direct maternally-imprinted transcription [26]. The dCas9-interacting *IGF2* promoter complexes were precipitated using a Cas9 antibody. We detected specific enrichment of the *IGF2* promoter DNAs in the precipitated chromatin complex (Figure 1B), with the strongest PCR signal seen at Site I, followed by Site II, regions that are located near promoters 2, 3 and 4 in the *IGF2* gRNA group. No enrichment was detected in the random gRNA (gCT) and dCas9 vector control groups (Vector), indicating successful precipitation of the targeted *IGF2* promoter chromatin complexes using this Cas9 immunoprecipitation approach. No signal was detected at the non-target Site III located near the non-imprinted promoter P1. Using small RNA library sequencing, we identified miR483, a well-defined oncogenic miRNA, as an RNA that interacted with the *IGF2* promoter complex.

We then used quantitative PCR to confirm the binding of miR483 to the *IGF2* promoters. Cells were transfected with dCas9-*IGF2*gRNA (gRNA), dCas9 control (Vector) and dCas9-control gRNA (gCT). After immunoprecipitation, quantitative PCR was used to measure the abundance of miR483 in the *IGF2* promoter chromatin complex. Using this assay, we confirmed the enrichment of miR483 in the Cas9 immunoprecipitated *IGF2* DNA complex (Figure 1C). There was no detectable signal of miR483 in the vector control or in the random gRNA control (gCT) groups. U6 is a non-coding small nuclear RNA (snRNA) used as the internal control in the miRNA quantitation kit. As expected, there was no enrichment of U6 snRNA in three groups. The specific binding of miR483 to *IGF2* promoters suggest a role for this microRNA in the regulation of the gene.

### Synthetic miR483 interacts with the *IGF2* promoter

**Figure 2:**
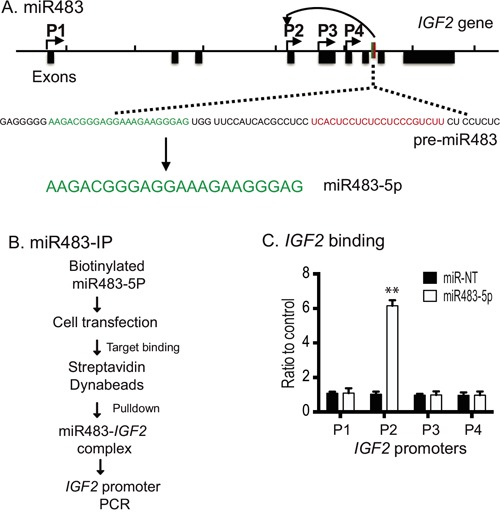
Biotinylated miR483 binds to the *IGF2* promoter **A**. Location of miR483-5p and its precursor in the *IGF2* locus. P1-P4: *IGF2* promoters. Pre-miR483 is transcribed from the *IGF2* intron 7. **B**. The biotinylated miR483 precipitation assay. After transient transfection, the biotin-miR483-5p interacting chromatin complex was pulled down by streptavidin beads. The presence of the *IGF2* promoter in the precipitated chromatin complex was measured by PCR. **C**. Binding of biotin-miR483-5p to the *IGF2* promoter. Real-time PCR was performed to quantitate the binding of miR483 to the *IGF2* promoter. * p<0.01 as compared with the random miRNA control (miR-NT).

The miR483 precursor (pre-miR483) is derived from *IGF2* intron 7 by cleavage by the Dicer ribonuclease; this produces both the mature sense miRNA (miR483-5p) and the mature antisense miRNA (miR483-3p) (Figure 2A). To confirm that the miRNA binds to the *IGF2* promoters, we synthesized miR483 and performed a miRNA-affinity precipitation assay (Figure 2B) [31]. We transfected tumor cells with biotin-labeled miR483-5p and random miRNA (miR-NT). The miRNA-interacting chromatin DNA was precipitated by streptavidin beads and analyzed by PCR. Using this assay, we demonstrated specific binding of the transfected biotin-miR483 to the imprinted *IGF2* promoter P2 (Figure 2C). No interaction of miR483 was observed with the non-imprinted *IGF2* promoter P1 or with the imprinted promoters P3 or P4. As expected, the biotinylated miR-NT control did not bind to *IGF2* promoter DNA. The fact that miR483 binds to the most upstream imprinted *IGF2* promoter P2 suggests that it may participate in the regulation of *IGF2* imprinting.

### Upregulation of *IGF2* by miR483-5p

**Figure 3:**
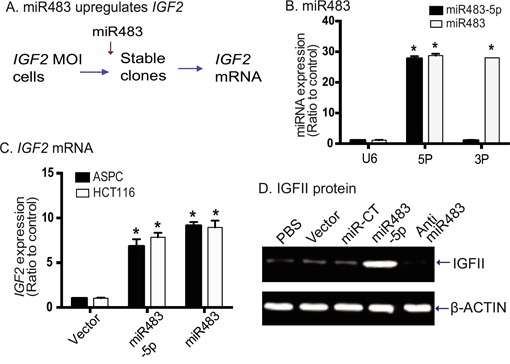
miR483 upregulates IGF2 **A**. Strategy to examine the role of miR483 in controlling *IGF2* allelic expression. MOI: cells with maintenance of *IGF2* imprinting. **B**. Expression of virally transfected miR483 in ASPC cells. Cells were transfected with miR483, miR483-5p, and random miRNA. Expression of miR483-5p, miR483-3P and control U6 were quantitated by qPCR. 5P: miR483-5p; 3P: miR483-3P. * p<0.01 as compared with the U6 control. **C**. Quantitation of *IGF2* expression in ASPC clones transfected with the empty vector, miR483-5p (5P), and miR483. * p<0.01 as compared with the vector control cells. **D**. Western blot of IGF-II protein. miR-CT: miRNA random control; Anti-miR483: miR483-5p inhibitor.

miRNAs have been reported to be able to modify expression and imprinting status of several genes [32, 33]. As miR483 binds to the *IGF2* promoter, we were interested in examining if the miRNA was able to control the activity of this promoter. We used lentiviral vectors to transfect miR483 precursor (pre-miR483) and miR483-5p into two tumor cell lines (ASPC and HCT116) that maintain normal *IGF2* imprinting [25]. After puromycin selection, stable clones were collected and used for the analysis of gene expression (Figure 3A).

We first confirmed the expression of pre-miR483 and miR483-5p in the transfected stable cells. After reverse transcription, using Q-PCR we found that miR483-5p was increased ˜27-fold in both the miR483 and 483-5P transfected cells (Figure 3B). miR483-3P was increased ˜26-fold in the miR483 group. The expression of the control U6 was not altered in either group.

We then used Q-PCR to quantitate *IGF2* expression in miR483 transfected cells. We found that both miR483 and miR483-5p upregulated the expression of the endogenous *IGF2*. In both tumor cell lines (ASPC, HCT116), we detected a ˜9-fold increase in *IGF2* mRNA transcripts in miRNA-transfected cells over that in the vector-control cells (Figure 3C). Similarly, using Western blot we found that miR483-5p increased IGF-II protein in treated cells (Figure 3D). These data suggest that miR483 activates *IGF2* expression, as previously reported [34].

### miR483 enhances tumor colony formation and cell proliferation

**Figure 4:**
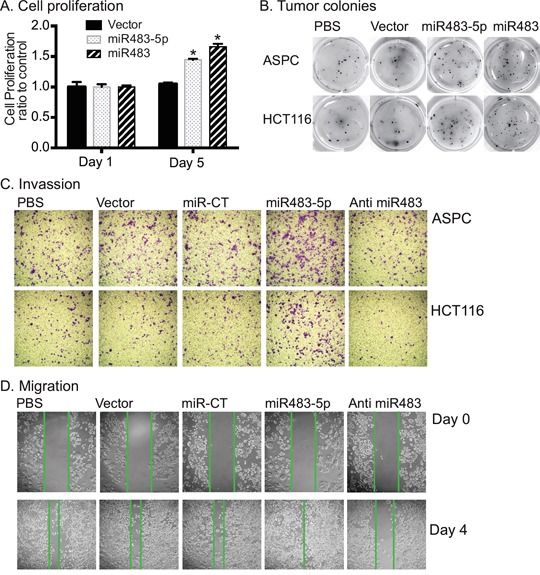
miR483 promotes the formation of tumor colony **A**. Cell proliferation. After miR483 transfection, cells were collected for analysis of cell proliferation using the MTT assay. * p<0.01 as compared with the vector control cells. **B**. Tumor colonies as measured by the Metri-gel assay. ASPC cell colonies were stained on day 15 and HCT116 cells on day 12. **C**. Cell invasion. Cells invaded through the collagen-coated membrane of the transwell were stained 16 hrs for ASPC and 24 hrs for HCT116. miR-CT: miRNA random control; Anti-miR483: miR483-5p inhibitor. **D**. Migration of ASPC cells. Cell migration was measured by scratch assay. Note the increased cell migration in the miR483-5p treated cells and the reduced migration in the miR483-5p inhibitor group.

IGF-II is an important mitogen that is associated with cell proliferation and tumor growth [35, 36]. We examined if the miR483-mediated upregulation of *IGF2* would affect cell proliferation in transfected tumor cells. In ASPC and HCt116 tumor cells, both miR483-5p and miR483 significantly enhanced cell growth by day 5 of culture (Figure 4A).

We also examined the effect of miR483 on tumorigenesis using a soft agar colony-forming assay. As seen in Figure 4B, miR483-5p and miR483 caused ˜2.5-3 fold more tumor colonies than did the empty vector control group. Similarly, miR483-5p also increased cell invasion (Figure 4C) and migration (Figure 4D, Supplementary Figure 2) in both ASPC and HCT116 cells. Inhibition of miR483 by a synthetic inhibitor (anti miR483-5p) decreased invasion and migration of tumor cells (Figures 4C, 4D). Thus, induced expression of miR483 increased proliferation and colony formation in both ASPC and HCT116 cancer cell lines.

### Activation of the maternal *IGF2* allelic by miR483

**Figure 5:**
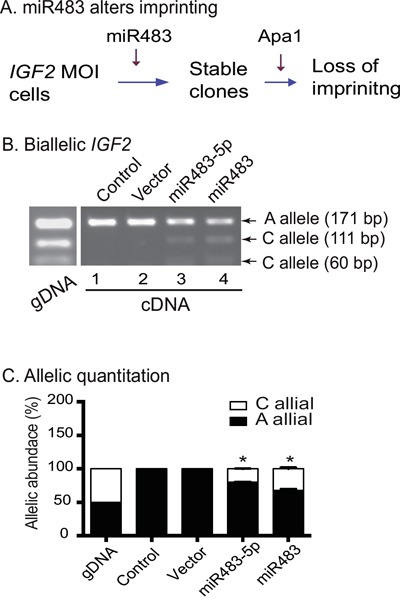
miR483 induces loss of *IGF2* imprinting **A**. Schematic diagram of *IGF2* imprinting in miR483-transfected cells. Allelic expression of *IGF2* was examined by polymorphic restriction enzyme Apa1. **B**. Biallelic expression of *IGF2* in miR483-transfeced cells. gDNA: genomic DNA. Note that both the “A” and “C” alleles are expressed in miR482-5P and miR483 cells. **C**. Quantitation of the two parental alleles. After separation by polymorphic restriction enzyme Apa1, the “A” and “C” alleles were quantitated by densitometric scanning. * p<0.01 as compared with control cells.

We next examined whether miR483 upregulated *IGF2* expression by altering the imprinting or by increasing expression from the non-imprinted allele. We used the polymorphic restriction enzyme Apa1 to distinguish the two parental alleles for imprinting analysis (Figure 5A).

In the untreated control and the vector cells, genomic DNA (gDNA) contained both the “A” and “C” alleles. In cDNAs, only the “A” allele product (171 bp) was detected (Figure 5B, lanes 1-2), exhibiting typical mono-allelic expression. In both the miR483 and miR483-5p clones, however, we found that the “C” allele products (111 bp and 60 bp) were also detected (lanes 3-4). These data suggest that the imprinted “C” allele was reactivated by miR483 and miR483-5p, leading to the relaxation or loss of *IGF2* imprinting.

Allelic quantitation also showed that the untreated control and the empty vector control cells expressed only the “A” allele. In the miR483- and miR483-5p cells, the “C” allele was activated, accounting for 20% to 30% of total *IGF2* mRNA transcripts (Figure 5C). These data suggest that induced expression of miR483 turned *IGF2* maintenance of imprinting (MOI) cancer cells into LOI cells.

### miR483 induces epigenetic modifications of *IGF2* promoters

**Figure 6:**
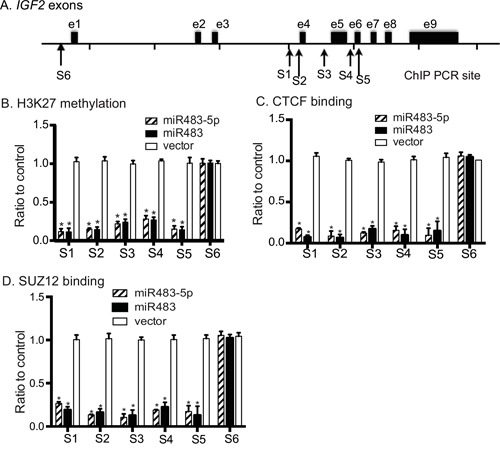
Epigenetic modifications in the *IGF2* promoters **A**. Schematic diagram of the *IGF2* genes and the location of PCR primers. **B**. Quantitation of Histone 3-K27 methylation. All data are obtained from three independent experiments and are presented as the relative values after normalization over the untreated cells (control). * p<0.01 as compared with control. **C**. Binding of CTCF to the *IGF2* promoters. All data shown are mean ± SEM from three independent experiments. * p<0.01 as compared with control. **D**. Binding SUZ12 to the *IGF2* promoters. All data shown are mean ± SEM from three independent experiments. * p<0.01 as compared with control.

*IGF2* imprinting is associated with specific monoallelic methylation of histone 3 lysine 27 (K27) in the gene promoters [37]. To explore the mechanism underlying the LOI, we used chromatin immunoprecipitation (ChIP) to examine whether miR483-mediated loss of *IGF2* imprinting is related to H3K27 methylation in the *IGF2* promoter region (Figure 6A).

We used quantitative PCR to measure the status of H3K27 methylation at the imprinted promoters (P2-P4)(Sites 1-5) and the non-imprinted promoter P1 (Site 6 as the control). As compared with the vector control group, cells transfected with miR483 and miR483-5p demonstrated a significant decrease in H3K27 methylation at all five sites (S1-S5) around the imprinted *IGF2* promoters (p<0.01, Figure 6B). No statistical difference was detected at the non-imprinted promoter P1 (S6).

A long range chromatin interacting complex containing CTCF and SUZ12 is required for specific monoallelic methylation of histone 3 lysine 27 (H3K27) in the *IGF2* promoters [37]. We examined whether miR483 affects the binding of CTCF and SUZ12 to the *IGF2* promoter region. Using ChIP, we noted a reduction of the binding of CTCF to all five sites across *IGF2* promoters P2-P4, which are imprinted (Figure 6C, sites S1-S5). The nonimprinted *IGF2* promoter 1 [26] was not affected by miR483 and miR483-5p (Figure 6C, site S6).

A similar pattern was also detected by ChIP assay of SUZ12, a docking factor of polycomb repressive complex 2 (PRC2) that is a critical factor in the regulation of *IGF2* imprinting [37, 38]. Induced expression of miR483 and mir483-P specifically reduced the binding of SUZ12 to the imprinting regulatory region of the *IGF2* promoter (Figure 6D). miR483 did not alter CTCF or SUZ12 mRNA expression in transfected cells (Supplementary Figure 3). Taken together, our data suggest that miR483 induces epigenetic modifications in the *IGF2* promoter complex, resulting in enhanced gene expression and loss of imprinting.

### miR483 activates the AKT pathway

**Figure 7:**
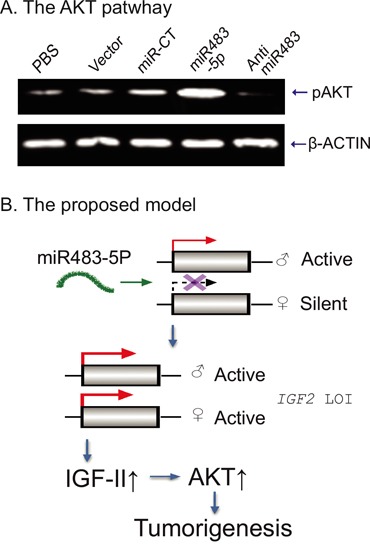
Putative model of the miR483-IGF2-AKT pathway **A**. The activated AKT pathway by miR483-5p. The phopho-AKT was detected by Western blot. **B**. The proposed model of miR483-5p in tumorigenesis. Normally, *IGF2* is maternally silenced. miR483-5p binds to the promoter and reactivates the maternal allele, leading to loss of *IGF2* imprinting (LOI). The overexpressed IGF-II growth factor induced activation of the AKT pathway in tumorigenesis.

Since IGF-II stimulates the PI3-K/AKT signaling cascade to promote tumor growth, we used Western blotting to quantitate phospho-AKT (pAKT). We found that induced expression of miR483-5p led to an increase in pAKT. Treatment of ASPC tumor cells with the miR483 inhibitor decreased pAKT (Figure 7A).

## DISCUSSION

Although loss of *IGF2* imprinting has been extensively reported in a variety of human malignancies, the molecular mechanisms underlying this tumor-specific dysregulation remain to be elucidated. Using a Cas9 immunoprecipitation assay, we identified miR483, an *IGF2* intronic microRNA, as a regulatory component in the *IGF2* promoter complex. miR483 is well-defined oncogenic microRNA that is overexpressed in a variety of human tumors [39–42]. In this study, we found that miR483 was able to bind to *IGF2* promoter P2. Induced expression of miR483 led to the activation of the normally imprinted allele. miR483 epigenetically upregulated *IGF2* by reducing promoter suppression mediated by histone H3 lysine 27 methylation. This is the first example of a microRNA serving as an important modulator of genomic imprinting in tumors.

MiRNAs regulate tens or hundreds of target genes [43] that are involved in a number of biological processes, including cell proliferation, cell death, stress resistance, and differentiation [44, 45]. It is generally agreed that miRNAs regulate gene expression at the post-transcriptional level by base pairing with the target sequence of the 3'-untranslated region, leading to mRNA cleavage and translational repression [46, 47]. However, whether miRNAs directly regulate their target genes at the transcriptional level, particularly through promoter binding regulation, has not been explored. In this study, we provide the first evidence that miR483 directly binds to the first upstream imprinted promoter (P2) of the *IGF2* gene, where it participates in the regulation of allelic expression. Upon binding to the promoter, it reduces the binding of two important imprinting regulators, SUZ12 and CTCF, and decreases the H3K27 methylation suppression mark in the promoter. This represents a novel method of participating in the control of *IGF2* imprinting.

Several chromatin factors have been implicated in the regulation of imprinting. The polycomb repressive complex 2 (PRC2), composed of core proteins *SUZ12* (suppressor of zeste 12), *EED* (embryonic ectoderm development) and *EZH2* (enhancer of zeste homologue 2), catalyzes the di- and tri-methylation of histone H3 at lysine 27 [48–50]. Our previous studies have demonstrated a direct role for PRC2 [37, 51–53], particularly its docking factor *SUZ12* [25, 54], in the regulation of *IGF2* allelic expression in many tissues. SUZ12 is downregulated in the *IGF2* loss of imprinting (LOI) tumor cell lines as compared with that in the *IGF2* maintenance of imprinting (MOI) tumor cell lines [25]. In *IGF2* LOI tumor cell lines that express low levels of SUZ12, ectopic expression of SUZ12 restores normal *IGF2* imprinting [54]. Appropriate docking of PRC2 through SUZ12 is required for maintaining H3K27 methylation mediated by the PRC2 methytransferase EZH2 in the *IGF2* promoter [25]. In this study, however, we found that miR483 itself does not affect SUZ12 abundance (Supplementary Figure 2). Instead, miR483 significantly reduced the binding of SUZ12 to the *IGF2* promoter (Figure 6D). miR483 may inhibit allelic H3K27 methylation by preventing PRC2 docking. However, it is not known how this microRNA might interfere with the docking of SUZ12 to the *IGF2* promoter.

CTCF is another chromatin factor that actively participates in the regulation of the imprinted *IGF2* allele [37, 51–53]. CTCF acts as a tethering protein, serving as a molecular glue to secure long range intrachromosomal [37, 55] and interchromosomal [56] interactions. By simultaneously binding to the unmethylated imprinting control region (ICR) and *IGF2* promoters, CTCF orchestrates the formation of a long-range chromosomal loop [55, 57, 58]. This CTCF-mediated chromatin looping brings the ICR and the *IGF2* promoters into close contact, where the polycomb repressive complex 2 (PCR2) is recruited via docking factor SUZ12, inducing allelic H3K27 methylation and gene silencing [37, 51]. In this study, we showed that miR483 reduced the binding of CTCF to the *IGF2* promoter (Figure 6D. With the reduction in H3K27 methylation in the promoter, the suppressed allele becomes activated, leading to biallelic expression of *IGF2*.

Several long noncoding RNAs (lncRNAs) regulate their target genes by directly binding to their promoters and enhancers, including *Kcnq1ot1, IRAIN, RUNXOR, Xist*, and *HOTAIR* [59–62]. Unlike lncRNAs, miR483 is a very short RNA, and we do not know how such a short microRNA, while binding to the promoter, also affects the binding of two other chromatin binding factors, SUZ12 and CTCF. Does miR483 bind to *IGF2* promoter P2 using base pairing mechanism as it does when it binds to the 3’-UTR region? What is the specific binding sequence of miR483-5p? Does it interfere with SUZ12 and CTCF binding through direct competition or by recruiting other RNA-binding factors? Future studies are needed to address these important issues. In addition, we have previously demonstrated that silencing *IGF2* significantly reduced the growth of implanted human hepatocarcinomas and prolonged lifespan in animal model [42, 43]. Future studies are needed to address the specific role of the miR483-5p/IGF pathway in animal models.

In summary, using Cas9 immunoprecipitation we identified the oncogenic miR483 as a critical component in the regulatory complex of *IGF2* imprinting. After binding to *IGF2* promoter P2, miR483 decreases the binding of CTCF and SUZ12 and consequently reduces H3K27 methylation. By altering the epigenotype in the promoter, miR483 upregulates *IGF2* production by relaxing *IGF2* imprinting. The overexpressed IGF-II growth factor may then promote tumorigenesis through the IGF1R/AKT pathway (Figure 7B). These data demonstrate a new role of the oncogenic miR483 in promoting tumor growth.

## MATERIALS AND METHODS

### Cell lines and cell culture

Human pancreatic cancer cell line ASPC-1 and colon cancer cell line HCT116, purchased from ATCC (Manassas, VA), were selected for this study because *IGF2* remains imprinted (monoallelically expressed) in both cell lines [25]. ASPC cells were routinely maintained in RPMI-1640 medium (Sigma, MO) and HCT116 cells in McCoy's 5a medium (Fisher, CA) containing 10% (v/v) fetal bovine serum (Sigma, MO), 100 U/ ml of penicillin sodium and 100 μg/ml of streptomycin sulfate (Invitrogen, CA), at 37 °C in 5% CO2 air atmosphere.

### Cas9-gRNA guided chromatin immunoprecipitation

A Cas9-guided chromatin immunoprecipitation assay [68] was used to identify components that bind to a target gene DNA fragment (Wang et al, unpublished data). In this study, we constructed the Cas9-*IGF2* gRNA vector by cloning two *IGF2* promoter gRNAs into the Cas9-2xgRNA vector that contains a mutated Cas9 (dCas9) and the tandem U6 and H1 promoters (Supplementary Figure 1A). Specifically, two oligonucleotides covering guiding RNA (gRNA) from the *IGF2* promoters P2 and P3 (P2-Site I: 5’-GCCTTGCGTTCCCCAAAATT-3’ and P3-Site II: 5’-GTCGCCGGCTTCCAGGTAAG -3’) were synthesized and were inserted immediately downstream of the U6 and H1 promoters, respectively, followed by the Cas9-hairpin RNA-(T)5 sequence (Supplementary Figure 1B).

The Cas9-*IGF2* gRNA lentiviruses were produced in 293T cells as previously described [60, 62, 63]. The viral supernatants were filtered through a 0.45-μm filter, concentrated by a PEG-IT kit (SBI, CA), aliquoted and stored in a −80°C freezer. An aliquot of the Cas9-*IGF2*gRNA lentivirus was used to transfect pancreatic cancer ASPC cells. After transfection, cells were selected by puromycin and collected for immunoprecipitation following the method as described previously [37, 64]. Briefly, cells were fixed with 1% formaldehyde and sonicated for 180 s (10 s on and 10 s off) on ice with a Branson sonicator with a 2-mm microtip at 40% output control and 90% duty cycle settings. The sonicated chromatin DNAs containing Cas9-gRNA-*IGF2* promoter complex were immunoprecipitated with Cas9 antibody (#ab191468, Abcam, MA). After reversal of cross-linking and proteinase K treatment, the Cas9-bound chromatin DNA and RNA were released and subjected to miRNA/DNA/RNA sequencing and analyses.

To amplify the miRNAs that interacted with the *IGF2* promoter, the Cas9-immunoprecipitated RNAs were treated with DNase I. The 3’ and 5’ SR adaptors were ligated to RNAs following the protocol of the NEBNext Small RNA library Prep Kit (#E7330S, NEB, MA). After PCR amplification, the predicted PCR bands were cut and cloned into a pJET vector (Thermo Fisher Scientific, CA) for sequencing.

### Construction of miR483 and miR483-5p vectors

The miR483 expression vectors were constructed by cloning pre-miR483 (precursor miRNA) and miR483-5p (mature miRNA) into pGreenPuro vector (#SI505A-1, SBI, CA). For pre-miR483, a pair of oligonucleotides JH2031 (5’-GATCCGAGGGGGAAGACGGGAGGAAAGAAGGGAGUGGUUCCAUCACGCCUCCUCACUCCUCUCCUCCCGUCUUCUCCUCUCG-3’) and JH2032 (5’-AATTCGAGAGGAGAAGACGGGAGGAGAGGAGUGAGGAGGCGUGAUGGAACCACUCCCUUCUUUCCUCCCGUCUUCCCCCUCG-3’) were synthesized for cloning. For miR483-5p, a pair of oligonucleotides JH2033 (5’-GATCCAAGACGGGAGGAAAGAAGGGAGTTTTTTG-3’) and JH2034 (5’-AATTCAAAAAACUCCCUUCUUUCCUCCCGUCUUG-3’) were synthesized for cloning. For miR483-5P inhibiting, a pair of oligonucleotides JH3816 (5’-GATCCCTCCCTTCTTTCCTCCCGTCTTTTTTTTG-3’) and JH3817 (5’-AATTCAAAAAAAAGACGGGAGGAAAGAAGGGAGG-3’) were synthesized for cloning. For cloning, equal amounts of each pair of oligonucleotides (1 μl 20 μM) were denatured at 95 C for 2 min and reannealed by gradually cooling to room temperature. The annealed fragments were ligated into the EcoR1/BamH1 site under control of the U6 promoter in pGreenPuro vector (SBI, CA). Oligonucleotides used for vector construction are listed in Supplementary Table 1. For the anti miR483-5p (the synthetic inhibitor), a pair of oligonucleotides JH3820 (5’-GATCCGAGAGGAGAAGACGGGAGGAGAGGAGTGAGGAGGCGTGATGGAACCACTCCCTTCTTTCCTCCCGTCTTCCCCCTCTTTTTTG-3’) and JH3821 (5’-AATTCAAAAAAGAGGGGGAAGACGGGAGGAAAGAAGGGAGTGGTTCCATCACGCCTCCTCACTCCTCTCCTCCCGTCTTCTCCTCTCG-3’) were synthesized to construct the vector (Supplementary Table 2).

The miR483 lentiviruses were produced in 293T cells and ASPC cells were transfected with viral supernatants in fresh medium containing 5 mg/ml polybrene (Sigma, MO). Three days after transfection, cells were selected with fresh medium containing 2 μg/ml puromycin. Stable cells were collected for imprinting and expression analysis.

### miRNA-affinity binding precipitation

A miRNA-affinity binding precipitation assay was performed to capture the genomic DNAs that interacted with miR483. Cells were plated in 6 well plates at 1 × 10^5^ cells/plate for 16 h and were transfected with biotin-miR483-5p or biotin-control RNA (20 pmol/well) using lipofectamine 2000 and OPTI-MEM I reduced serum medium (Invitrogen, CA). Twenty-four hours following transfection, cells were lysed in nuclei lysis buffer and sonicated. DNAs that interacted with biotin-miR483-5p were pulled down using streptavidin beads according to the manufacturer's protocol (Invitrogen, CA). The enriched miR483-5p chromatin DNA complex was analyzed by PCR using primers from the *IGF2* promoter (Supplementary Table 2).

### RT-PCR

Total RNA was extracted by TRIzol reagent (Sigma, MO) from cells and stored at −80 °C. The following primers were used for PCR: 1). *β-ACTIN* forward J880 and reverse J881; 2). *IGF2* (covering Apa1 site) forward JH2505 and reverse JH2506 (Supplementary Table 3). RT-PCR reaction was performed with a Bio-Rad Thermol Cycler. The amplification of *IGF2* was achieved by PCR of 1 cycle at 95°C for 5 min, 33 cycles at 95°C for 20s, 62°C for15s and 72°C for 15 s, and 1 cycle at 72 °C for 10 min.

Quantitative real-time PCR was performed using SYBR GREEN PCR Master (Applied Biosystems, USA) as previously described [25, 65]. The threshold cycle (Ct) values of target genes were assessed by quantitative PCR in triplicate using a sequence detector (ABI Prism 7900HT; Applied Biosystems) and were normalized over the Ct of the β-ACTIN control.

### Imprinting analysis of the *IGF2* gene

The status of *IGF2* imprinting in tumor cells was evaluated by RT-PCR as previously described [25, 54]. The *IGF2* mRNA covering the polymorphic Apa1 site was amplified by PCR primers JH2505 and JH2506, using 1 cycle at 95°C for 5 min, 33 cycles at 95°C for 20s, 62°C for 15s and 72°C for 15 s, and 1 cycle at 72 °C for 10 min. PCR products were digested with Apa1 to distinguish the two parental alleles and were separated on a 3% agarose gel. The digested and undigested alleles were both quantitated using ImageJ software. Allelic expression was quantitated using the following formula after normalizing over the gDNA control: (C1+C2)/(A+C1+C2)_(cDNA)_/(C1+C2)/(C1+C2+A)_(gDNA)_ x 100%. (A: undigested allele; C1+C2: the digested allele).

### Tumor colony forming assay

The tumor clonogenic assay was performed as previously reported [64]. Briefly, 0.5% and 0.25% agarose (Sigma, MO) were prepared with sterile H_2_O and stored in a 4°C refrigerator. DMEM culture medium containing 10% FBS was prepared and kept in a 37°C water bath. Then 60 μl of 0.5% agarose and 540 μl DMEM were mixed and layered onto 24-well plates as the base agar. Cells were digested, centrifuged and resuspended in DMEM to form single cell suspensions. Cells were adjusted to 2×10^3^ cells/ml in 0.25 ml DMEM, mixed with 0.25 ml 0. 25% agarose, and added onto the base agar. The plates were incubated at 37°C, 5% CO_2_ until visible colonies (N > 50/group) were formed. After ˜2 weeks, colonies were visualized by staining with 5 mg/ml MTT (3-(4,5-dimethyl-thiazol-2-yl)-2,5-diphenyltetrazolium bromide) (Sigma, MO) for 3 hours, and then the colonies were photographed and counted. Cloning forming efficiency (CFE) was defined as the number of colonies / number of inoculated cells×100%.

### Cell proliferation assay

Cell survival was measured using the MTT assay [66, 67]. Briefly, cells (1×10^4^/well) were plated onto 96-well plates and were incubated with 20 μl 5 mg/ml MTT (Sigma, MO) per well at 37 °C for 4 h. The absorbance was measured at 490 nm using a microplate reader (Bio-TEK Instruments, USA). Cell viability (%) was calculated based on the following equation: Cell viability (%)1/4(Asample/Acontrol) x 100%, where Asample and Acontrol represent the absorbance of the sample and control wells, respectively.

### Chromatin immunoprecipitation (ChIP)

ChIP assays were performed with a ChIP assay kit (Millipore, NY) by following the protocol provided by the manufacturer with slight modifications as previously described [51]. Briefly, 5 million cells were fixed with 1% formaldehyde and then sonicated for 180 s (10 s on and 10 s off) on ice using a Branson sonicator with a 2-mm microtip at 40% output control and 90% duty cycle settings. The sonicated chromatin was immunoprecipitated with specific antibodies to CTCF, SUZ12, and dimethyl-H3-K27 (lysine 27 of histone H3)(Cell Signaling, MA). Anti-IgG was used as the ChIP control in parallel with testing samples. ChIP DNAs were quantitated by qPCR using target gene primers (Supplementary Table 2). For comparison, the ChIP data are presented as relative values by normalizing to PCR signals of input DNA (i.e. ratio of the ChIP over the input).

### Statistical analysis

All experiments were performed in triplicate, and the data are expressed as mean ± SD. Data were analyzed using SPSS software (version 16.0; SPSS, Inc., IL). Student's t-test or one-way ANOVA (Bonferroni test) was used to compare statistical differences for variables among treatment groups. Results were considered statistically significant at p<0.05.

## SUPPLEMENTARY MATERIALS FIGURES AND TABLES


