# Supplementary material for: CRISPR Cas9-guided chromatin immunoprecipitation identifies miR483 as an epigenetic modulator of IGF2 imprinting in tumors
Source: Oncotarget. 2016 Jul 29;8(21):34177–90. doi: 10.18632/oncotarget.10918 (PMC5470959; doi:10.18632/oncotarget.10918)
Supplement: Supplementary file 1 [file oncotarget-08-34177-s001.pdf]

CRISPR Cas9-guided chromatin immunoprecipitation identifies miR483 as an epigenetic modulator of *IGF2* imprinting in tumors

SUPPLEMENTARY FIGURES AND TABLES

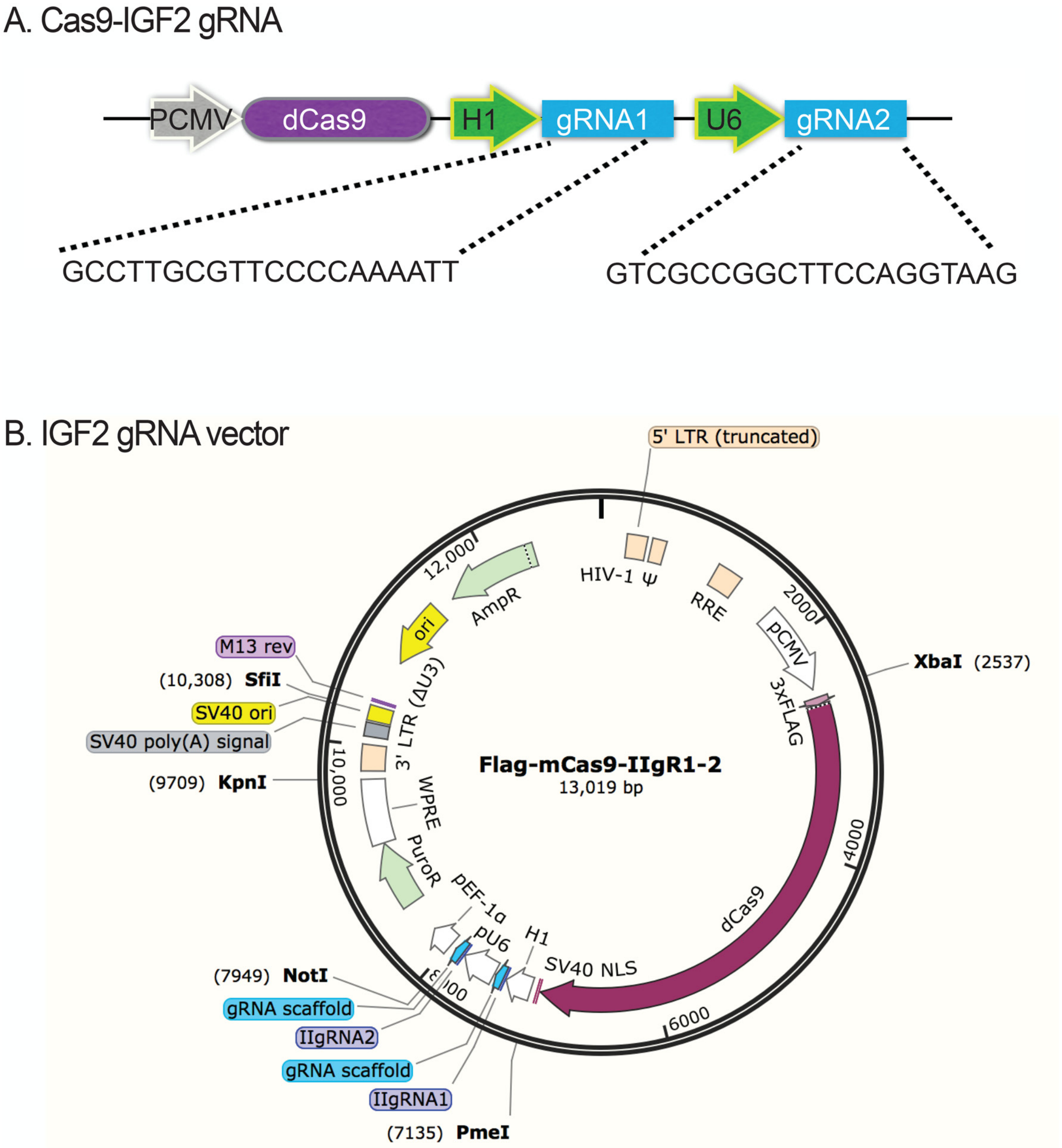

Supplementary Figure 1: Construction of CRISPR Cas9-IGF2 gRNA vectors. A. Diagram of the CRISPR Cas9-IGF2 gRNA vector. dCas9: mutated Cas9; gRNA: IGF2 guiding RNA; H1: RNA polymerase III H1 promoter; U6: RNA polymerase III U6 promoter; pCMV: CMV promoter. **B.** The CRISPR Cas9-IGF2 gRNA lentiviral vector. Two IGF2 gRNAs driven by RNA polymerase III H1 and U6 are inserted in the downstream of dCas9.

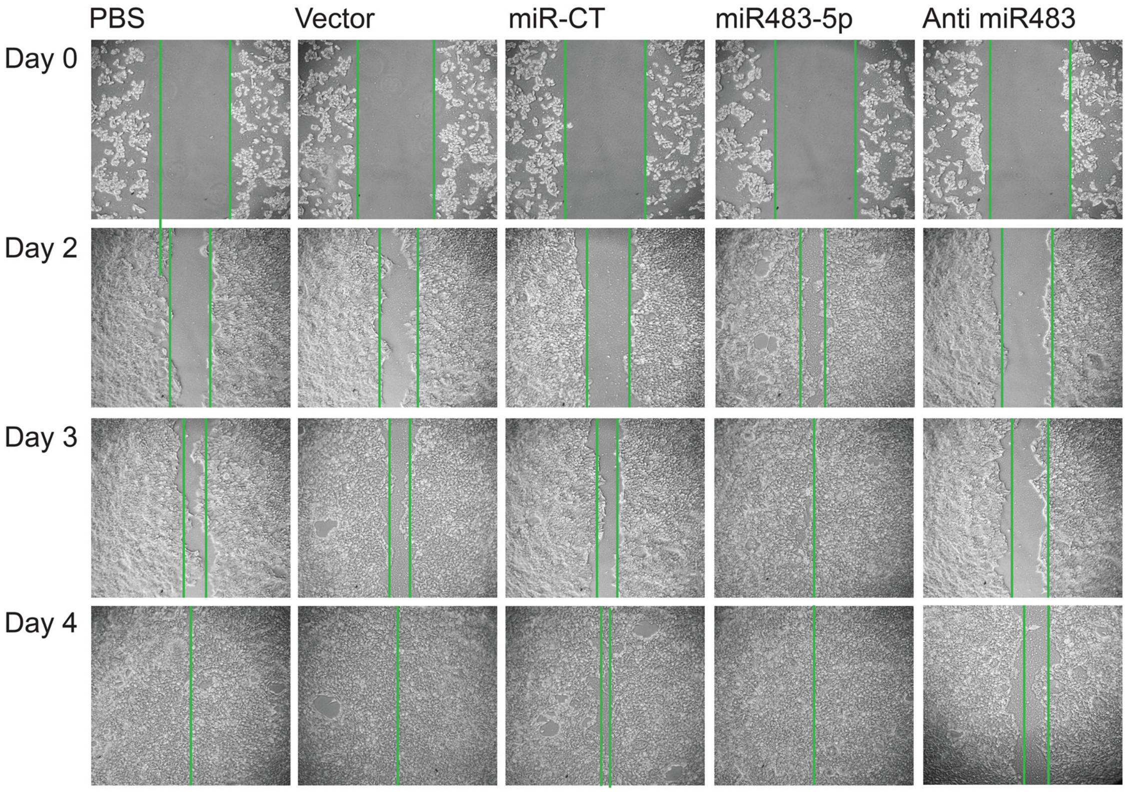

Supplementary Figure 2: Migration of ASPC cells. HCT116 colon cancer cells were treated with PBS, vector, miR483-5p, and miR483-5p inhibitor, respectively. The migration of cells was measured by the scratch assay. Note the increased cell migration in the miR483-5p treated cells and the reduced migration in the miR483-5p inhibitor group.

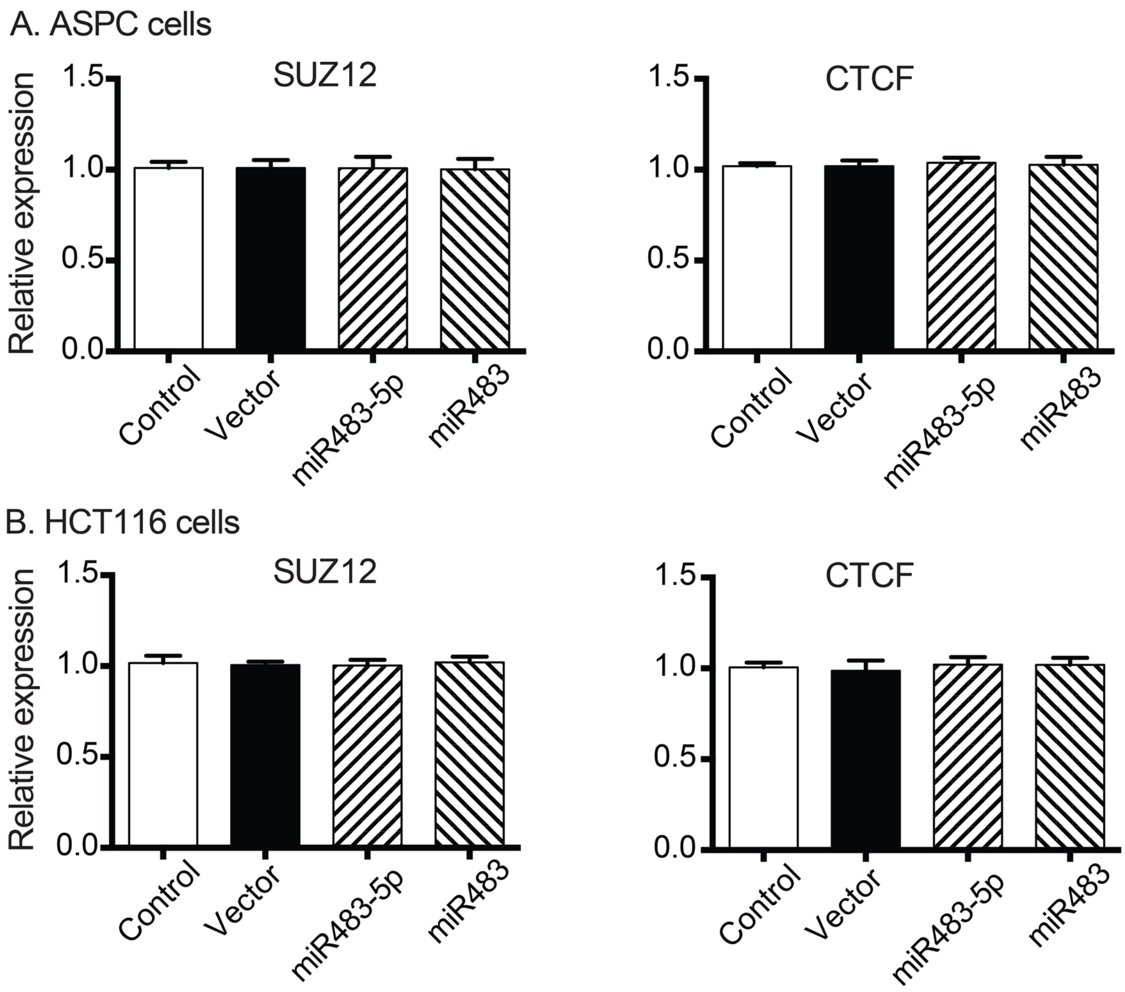

Supplementary Figure 3: miR483 did not affect the expression of IGF2 imprinting regulatory factors CTCF and SUZ12. A. Expression of SUZ12 and CTCF in miR483-transfected ASPC cells. **B.** Expression of SUZ12 and CTCF in miR483-transfected HCT116 cells.

Supplementary Table 1: Primers used for vector cloning

JH2031	GATCCGAGGGGGAAGACGGGAGGAAAGAAGGGAGTGGTTCCATC ACGCCTCCTCACTCCTCTCCTCCCGTCTTCTCCTCTCG	miRNA-483
JH2032	AATTCGAGAGGAGAAGACGGGAGGAGAGGAGUGAGGAGGCGTG ATGGAACCACTCCCTTCTTTCCTCCCGTCTTCCCCCTCG	
JH2033	GATCCAAGACGGGAGGAAAGAAGGGAGTTTTTTG	miRNA483-5P
JH2034	AATTCAAAAAACTCCCTTCTTTCCTCCCGTCTTG	
JH3820	GATCCGAGAGGAGAAGACGGGAGGAGAGGAGTGAGGAGGCGTGA TGGAACCACTCCCTTCTTTCCTCCCGTCTTCCCCCTCTTTTTTG	anti-miRNA483
JH3821	AATTCAAAAAAGAGGGGGAAGACGGGAGGAAAGAAGGGAGTGGT TCCATCACGCCTCCTCACTCCTCTCCTCCCGTCTTCTCCTCTCG	
JH3816	GATCCCTCCCTTCTTTCCTCCCGTCTTTTTTTTG	anti-miRNA483-5P
JH3817	AATTCAAAAAAAAGACGGGAGGAAAGAAGGGAGG	
JH3915	TAGTAATGAGTTTAAACAAGGTCGGGCAGGAAGAGGGCCT	CAS9-F1
JH3916	CAATTTTGGGGAACGCAAGGCGGTGTTTCGTCCTTTCCACAAG	CAS9-R1
JH3917	GCCTTGCGTTCCCCAAAATTGTTTTAGAGCTAGAAATAGCAAGTT	CAS9-F2
JH3918	ACCTTACCTGGAAGCCGGCGACGGATCCAAGGTGTCTCATACAG	CAS9-R2
JH3919	GTCGCCGGCTTCCAGGTAAGGTTTTAGAGCTAGAAATAGCAAGTT	CAS9-F3
J441	CAACTTCTCGGGGACTGTGGGCGAT	CAS9-R3
JH3920	GCACTGCATCTAGGCAGCGCGGTGTTTCGTCCTTTCCACAAG	CAS9 CT-R1
JH3921	GCGCTGCCTAGATGCAGTGCGTTTTAGAGCTAGAAATAGCAAGTT	CAS9 CT-F2
JH3922	AAGCAAATGTGATAAGAGCAGGATCCAAGGTGTCTCATACAG	CAS9 CT-R2
JH3923	TGCTCTTATCACATTTGCTTGTTTTAGAGCTAGAAATAGCAAGTT	CAS9 CT-F3

Cas9 CT: Cas9 random gRNA vector.
F: Forward primer; R: Reverse primer.

Supplementary Table 2: Primers used for the ChIP assay

JH3778 (TL58)	ATCCATACAAGGAGGTGGGAACCAG	101bp	Site 1	P2/−0.5 kb
JH3779 (TL59)	AACCGGGAGCCCTGGACCATCCCGT			
JH3780 (T150)	TCTGTCTCCTACGAAGTCCCCAGAG	114kb	Site 2 (P2)	P2/0 kb
JH3781 (T151)	GAAGCCCTCCCTGTCCACGTCCTGA			
JH3783 (T152)	TGCCTGCCCGGAGACCCCAGCTCAC	86bp	Site 3	P2/P4 0.83 kb
JH3784 (T153)	CGCAGAGCGCCAAGGCCATGCTGAA			
TL38	AAGAGACTGGCTGGGAGGAGGGAGA	125bp	Site 4	P4/−0.15 kb
TL39	CTCAGCTTTTATGTGTGAGCCGACT			
T140	CAGCCTGGGCCCCTGCAGCTGTGGAT	103bp	Site 5-F<br>Site 5-R	P4/+1.4 kb
5104	CCAGTTCCTTCCATTTGCAAGAAGC			
5077	TGCACCTTTCCTGAGAGCTCCAC	79bp	Site 6 (P1)	P1/0 kb
5076	TCTGCAGCCACAGCCCATGCCAGC			
JH3996	CTGCTCTCCGGCGGAGCT	118bp	P3	P3/0 kb
JH3997	CTTGCCCGATGGAGGCGCT			
JH3998	AGGCACTGACCAGCCTGCA	114bp	P4	P4/-0 kb
JH3999	AGCGAGCCTTCTGCTGAGC			
JH3901	OGAUGCUACGGUCAAUGUCUAAG		miRNA-random control	
JH3902	OAAGACGGGAGGAAAGAAGGGAG		miRNA-483 5P	

P: Promoter

Supplementary Table 3: Primers for IGF2 imprinting and miRNA483 expression

JH2505	CTTGGACTTTGAGTCAAATTGGCCT	171bp	IGF2 Apa1 site
JH2506	GAGGAGCCAGTCTGGGTTGTTGCTA		
J880	CAGGTCATCACCATTGGCAATGAGC	135bp	β-ACTIN
J881	CGGATGTCCACGTCACACTTCATGA		
JH3536	AAGACGGGAGGAAAGAAGGGAG		miRNA483 5P-F
JH3537	TCACTCCTCTCCTCCCGTCTT		miRNA483 3P-F
